# Potential pharmacological mechanisms of tanshinone IIA in the treatment of human neuroblastoma based on network pharmacological and molecular docking Technology

**DOI:** 10.3389/fphar.2024.1363415

**Published:** 2024-03-11

**Authors:** Ning Tang, Yan Wang, Jiarui Miao, Yang Zhao, Yue Cao, Wentao Sun, Jingke Zhang, Hua Sui, Bing Li

**Affiliations:** ^1^ Department of Integrative Medicine, Liaoning University of Traditional Chinese Medicine Xinglin College, Shenyang, China; ^2^ Department of Integrative Medicine, Dalian Medical University, Dalian, China; ^3^ Department of Acupuncture and Massage, Liaoning University of Traditional Chinese Medicine Xinglin College, Shenyang, China

**Keywords:** neuroblastoma, tanshinone IIA, network pharmacology, molecular docking, gene ontology

## Abstract

Tanshinone IIA (Tan-IIA) is the main bioactive component of Chinese herbal medicine salvia miltiorrhiza (Danshen). Sodium sulfonate of Tan-IIA is widely used in the treatment of cardiovascular and cerebrovascular diseases. Tan-IIA also has inhibitory effects on tumor cells such as gastric cancer, but its therapeutic effect and mechanism on human neuroblastoma have not been evaluated, so its pharmacological mechanism is systematically evaluated by the combined method of network pharmacology and molecular docking. PharmMapper and SwissTargetPrediction predicted 331 potential Tan-IIA-related targets, and 1,152 potential neuroblastoma-related targets were obtained from GeneCards, DisGeNET, DrugBank, OMIM and Therapeutic Target databases (TTD), 107 common targets for Tan-IIA and neuroblastoma. Through gene ontology (GO) functional annotation, Kyoto Encyclopedia of Genes and Genomesa (KEGG) pathway enrichment, protein-protein interaction (PPI) network and cytoHubba plug-in, 10 related signal pathways (Pathways in cancer, PI3K-Akt signaling pathway, Prostate cancer, etc.) and 10 hub genes were identified. The results of molecular docking showed that Tan-IIA could interact with 10 targets: GRB2, SRC, EGFR, PTPN1, ESR1, IGF1, MAPK1, PIK3R1, AKT1 and IGF1R. This study analyzed the related pathways and targets of Tan-IIA in the treatment of human neuroblastoma, as well as the potential anticancer and anti-tumor targets and related signaling pathways of Tan-IIA, which provides a reference for us to find and explore effective drugs for the treatment of human neuroblastoma.

## 1 Introduction

Neuroblastoma (NB) is the most common extracranial solid tumor in childhood and the most common cancer in infancy. It originates from the neural crest progenitor cells of the developing sympathetic nervous system, and includes ganglioneuroblastoma and ganglioneuroma, accounting for 15% of cancer mortality in children (Cheung and Dyer, 2013; Matthay et al., 2016). Neuroblastoma usually occurs in the paravertebral sympathetic ganglia or pelvic ganglia of the neck, chest and abdomen, as well as in the chromaffin cells of the adrenal medulla. Its etiological types are different in biological, genetic, clinical and morphological characteristics (Luksch et al., 2016). As a common solid tumor in children, NB usually affects preschool children, but studies have shown that in some cases it may also occur in adolescents and adults. Among children with NB, the survival rate has improved slightly over the past few years, but the mortality rate is still close to 50 per cent. Currently, the main treatment methods of neuroblastoma include surgery, radiotherapy, chemotherapy, bone marrow transplantation and so on, but the prognosis is poor (Maris et al., 2007; Maris, 2010), The risk of severe disability and chronic disease is still very high after treatment, and chemotherapy-related toxicity is often observed (Brizzolara et al., 2020). In this case, there is an urgent need to develop new therapeutic methods with high efficiency and low toxicity. Therefore, it is of great significance to deeply study the pathogenesis of NB and find the key targets of its treatment.

**FIGURE 1:**
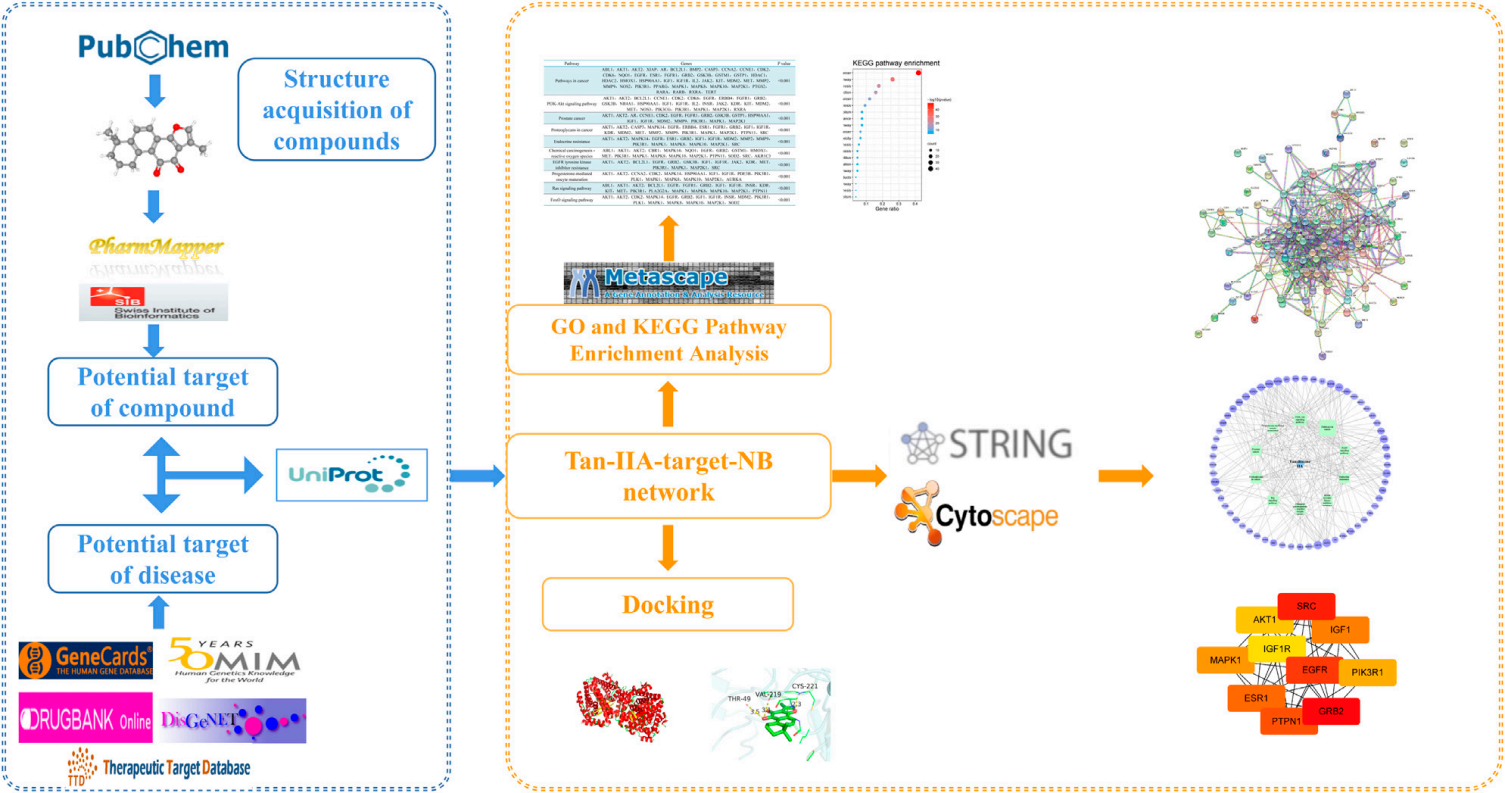
Flow chart of network pharmacology research.

Tanshinone IIA (Tan-IIA) is a natural diterpene quinone isolated from the root of salvia miltiorrhiza and is one of the most pharmacologically active components in salvia miltiorrhiza (Gao et al., 2012). In recent years, Tan-IIA has been used in animal and *in vitro* studies to treat cardiovascular diseases, postmenopausal syndrome, angina pectoris, myocardial infarction, hypertension, hyperlipidemia, acute ischemic stroke, chronic renal diabetes, Alzheimer’s disease and cancer (Liu et al., 2010; Ru et al., 2014; Lu et al., 2016; Kim et al., 2021), Tan-IIA sulfonate is also widely used in the treatment of cardiovascular and cerebrovascular diseases (Gao et al., 2012). In addition, previous studies have shown that Tan-IIA can effectively inhibit the proliferation of many kinds of tumor cells, and some studies have proved that Tan-IIA has a neuroprotective effect (Lu et al., 2016). Currently, studies on Tan-IIA in the treatment of NB have shown that it can inhibit the proliferation of tumor cells, but there is no study on the specific molecular mechanism of its effect. Therefore, based on the network pharmacology research method, the molecular targets and pathways of Tan-IIA in the treatment of NB were predicted and verified by molecular docking. The flow chart of the research route is shown in Figure 1.

## 2 Materials and methods

### 2.1 Materials

TCMSP (https://tcmsp-e.com/tcmsp.php) (Ru et al., 2014), PubChem (https://pubchem.ncbi.nlm.nih.gov/) (Kim et al., 2021), PharmMapper (http://www.lilab-ecust.cn/pharmmapper/) (Liu et al., 2010; Wang et al., 2016; Wang et al., 2017), SwissTargetPrediction (http://www.swisstargetprediction.ch/) (Gfeller et al., 2013; Daina et al., 2019), GeneCards (https://www.genecards.org/) (Stelzer et al., 2016), DisGeNET (https://www.disgenet.org/) (Pinero et al., 2021), DrugBank (https://go.drugbank.com/) (Wishart et al., 2018), OMIM (https://www.omim.org/) (Amberger and Hamosh, 2017), TherapeuticTargetDatabase (http://db.idrblab.net/ttd/) (Zhou et al., 2022), STRING (https://cn.string-db.org/) (Szklarczyk et al., 2021), Metascape (https://metascape.org/gp/index.html) (Zhou et al., 2019), jVeen (http://jvenn.toulouse.inra.fr/app/index.html) (Bardou et al., 2014), Cytoscape (https://cytoscape.org/) (Shannon et al., 2003), AutoDock 1.5.6 (https://autodock.scripps.edu/) (Zhang and Sanner, 2019), RCSB Protein Data Bank (http://www.rcsb.org/) (Burley et al., 2021), PyMOL (https://pymol.org/2/) (Swain et al., 2022).

### 2.2 Collection of pharmacokinetic information of Tan-IIA by TCMSP

TCMSP is a systematic pharmacology database providing information of traditional Chinese medicine about compounds, ADME-related (absorption, distribution, metabolism and excretion) characteristics, targets and diseases. We can find Chinese herbal medicines with potential biological effects through the database. We used ‘Tanshinone IIA’ as the key word and searched for its pharmacological and molecular properties in TCMSP.

### 2.3 Prediction of related targets of Tan-IIA

PubChem is a public database of small molecular bioactivity data, from which the related files of Tan-IIA are downloaded and uploaded to SwissTargetPrediction and PharmMapper databases to predict their molecular targets. By comparing the molecular similarity with the active compounds of Tan-IIA, the targets related to Tan-IIA were screened. The common potential drug targets predicted by the two databases were selected for further verification.

### 2.4 Prediction of related targets in human neuroblastoma

Acquisition of related targets of human neuroblastoma through GeneCards、DisGeNET、DrugBank、OMIM、TherapeuticTargetDatabase database. These databases contain rich and cutting-edge disease-related goals. All databases were searched with ‘Neuroblastoma’ as the keyword, and common genes related to human neuroblastoma were selected as candidate targets.

### 2.5 PPI network construction

UniProt protein database was used to standardize the disease target and the potential target of drug composition as GeneSymbol, and then the two were mapped to obtain the potential target of Tan-IIA to interfere with human neuroblastoma. The common targets between drugs and diseases were screened by jveen, and the co-expression targets were obtained.

STRING aims to collect and integrate information about all functional interactions between expressed proteins. The Tan-IIA-related goals and neuroblastoma-related goals obtained from the above steps were analyzed and compared. Then the overlapping genes of the target were extracted and introduced into STRING 11.5, and “*Homo sapiens*” was selected from the optional species, and the minimum interaction score 0.7 was high confidence score. Then the drug-disease target PPI network was obtained.

### 2.6 Enrichment analysis of gene function and pathway

The list of coexpressed target genes of Tan-IIA in the treatment of human neuroblastoma was inputted into the Metascape database, and the enrichment analysis of GO and KEGG was carried out.

### 2.7 Constructing Tan-IIA-targetpathway network

Through the information obtained in “2.5” and “2.6” steps, the Cytoscape3.8.2 software was used to establish the relationship between the active compound tanshinone IIA, candidate targets and the related KEGG pathway (drug-target-pathway network), to intuitively understand the interaction of each node. In addition, the first 10 hub genes were predicted using the cell Hubba plug-in in Cytoscape 3.8.2 and the Tan-IIA-target-pathway network was formed.

### 2.8 Molecular docking

Molecular docking can simulate the interaction between small molecular ligands and biological macromolecular receptors, and predict the binding mode and affinity between them, to select the leading drugs that can bind to the target and provide guidance for the reasonable optimization of drug molecular structure. It is of great significance for drug research and development. The top 10 gene targets of Tan-IIA- NB were selected and analyzed. The crystal structure of the corresponding gene target protein was downloaded from RCSB Protein Data Bank (PDB) database and preserved in pdb format. The 3D structure of Tan-IIA was obtained by PubChem database and converted into mol2 format file by Chem3D. Removal of water molecules and heteroatoms from receptors with AutoDock Tools 1.5.6 and PyMol 2.5.2, and hydrogen atoms and calculated charges were added, which were stored in pdbqt format (Shi et al., 2022). Then, the conformation of ligand-receptor binding was predicted by AutoDockTools 1.5.6. Binding energy is one of the results of molecular docking, which can be used to screen the potential of ligand-receptor binding, in which the docking result ≤ -5 kcal/mol can be considered to have binding activity between ligand and nuclear receptors (Yang et al., 2021). The conformation with the best binding energy was selected as the final conformation and saved as a pdb format file for imaging with PyMOL 2.5.2.

### 2.9 Experimental verification

#### 2.9.1 Cell culture and treatment

Human neuroblastoma SH-SY5Y cell line was cultured in DMEM complete medium containing 10% fetal bovine serum and 1% penicillin/streptomycin in 5% CO_2_ and 95% air humidification incubator. At 37°C, SH-SY5Y cells were adherent cells, and the medium was changed every day until the cells reached 60%–80% density. Digest with 0.25% trypsin solution, and then add DMEM to stop digestion, during passage, the cells were resuscitated to the appropriate density and transferred to the culture flask to continue culture.

#### 2.9.2 Cell proliferation assay

Human neuroblastoma cell SHSY-5Y viability was measured by MTT assay. One day before administration, SHSY-5Y cells in logarithmic growth phase were prepared into a single cell suspension with DMEM complete medium. 3,000 cells per well were inoculated into 96-well plate and cultured at 37°C in 5% CO_2_ cell incubator. After being treated with Tan ⅡA for 24 h, the supernatant was discarded after being incubated with 5 mg·mL^-1^ MTT solution 10 μL at 37°C for 4 h. DMSO 150 μL was added to each well. 10 min was shaken at low speed on the shaker, and the absorbance was determined by wavelength 570 nm with multi-function enzyme labeling instrument.

#### 2.9.3 Wound healing migration assay

SHSY-5Y was allowed to grow into full confluence in 6-well plates. A line of SHSY-5Y cells was then scraped away in each well using a pipette tip. Subsequently, cells were washed twice with PBS to remove detached cells. Fresh DMEM medium containing different concentrations of Tan ⅡA (0, 20 and 50 μmolL^-1^) was added to the scratched monolayers. Images were taken using an inverted microscope (Eclipse TS100, Nikon, Japan) after 12 h of incubation. The migrated cells were observed from three randomly chosen fields and quantified by manual counting. Inhibition percentage was expressed as percentage of the untreated cells (100%).

#### 2.9.4 Detection of lipid peroxide content in tumor cells

BODIPY-C11 581/591 is a fat-soluble fluorescent probe that can indicate the presence of lipid peroxides in living cells. SH-SY5Y cells were inoculated in 24-well plates and incubated at the concentration of 5 μM BODIPY-C11 581/591 in 5% CO_2_ incubator at 37°C for 30 min. Wash it gently with PBS twice at room temperature, and then use BODIPY-C11 probe to detect the concentration of iron ion and the level of lipid peroxidation under fluorescence microscope or confocal microscope. According to the quantitative analysis of the measured fluorescence signal, the excitation wavelengths of BODIPY-C11 are 581 nm (reduced, red) and 500 nm (oxidized, green).

## 3 Results and discussion

### 3.1 Pharmacokinetic information of Tan-IIA

The pharmacological and molecular properties of Tan-IIA are obtained using TCMSP (Table 1), including molecular weight (MW), low lipid-water partition coefficient (AlogP), hydrogen bond donor (Hdon), hydrogen bond receptor (Hacc), oral bioavailability (OB), Caco-2 permeability (Caco-2), blood-brain barrier (BBB) and drug-like properties (DL). Generally speaking, according to the parameter information and screening criteria of TCMSP database, the DL of effective drugs should be greater than 0.18. Clearly, Tan-IIA shows good drug similarity, which is worthy of further study.

**TABLE 1:** Pharmacological and molecular properties of Tanshinone IIA.

Name	MW	AlogP	Hdon	Hacc	OB (%)	Caco-2 (nm/s)	BBB	DL
Tanshinone IIA	294.37	4.66	0	3	49.89	1.05	0.70	0.40

### 3.2 Overlapping targets of Tan-IIA and NB

**FIGURE 2:**
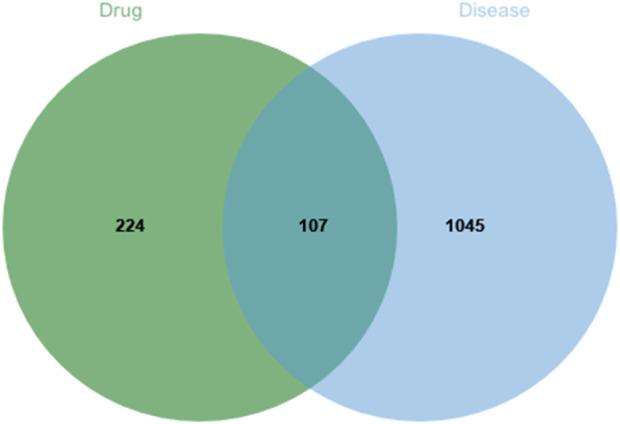
Intersection of Tanshinone IIA and Neuroblastoma (The blue circle represents the disease targets and the green circle represents the drug targets).

SwissTargetPrediction and PharmaMapper predicted 331 potential Tan-IIA related targets for further verification and obtained 1152 NB related targets from GeneCards, DisGeNET, DrugBank, OMIM and TTD databases. 107 overlapping genes of the target were identified as coexpression targets (Figure 2).

### 3.3 PPI network analysis

**FIGURE 3:**
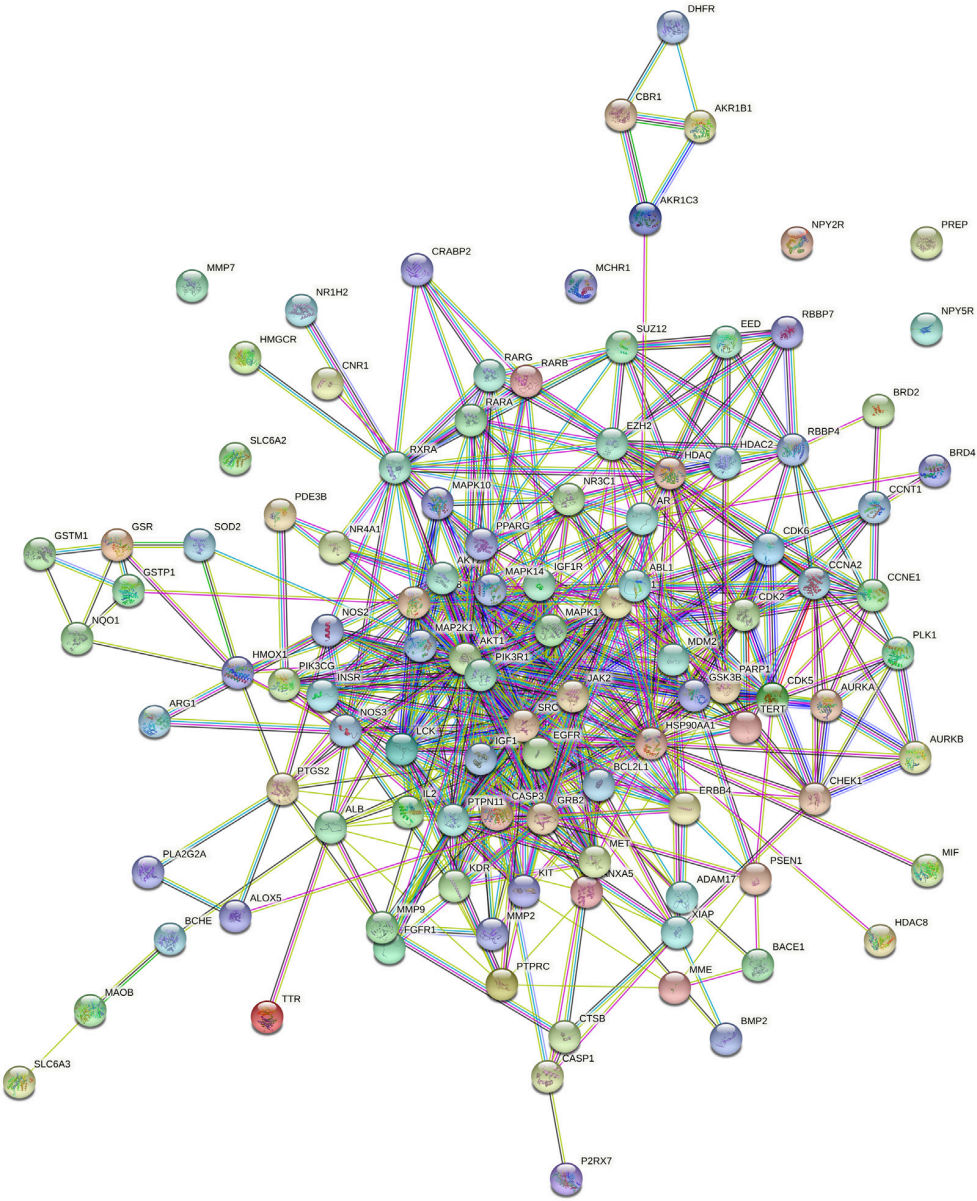
PPI network analysis diagram (nodes represent proteins, lines represent interactions).

The PPI network analyzed by STRING 11.5 is shown in Figure 3. The proteins in the PPI network are represented by circular nodes with protein 3D structure inside, and the lines between the nodes represent the interaction between proteins. The more lines, the stronger the relationship between the two. A total of 107 genes were input, of which 101 nodes interacted with each other, and the other six nodes (NPY2R, PREP, MMP7, NPY5R, MCHR1 and SLC6A2) were not associated with other nodes.

### 3.4 GO and KEGG pathways enrichment analysis

**FIGURE 4:**
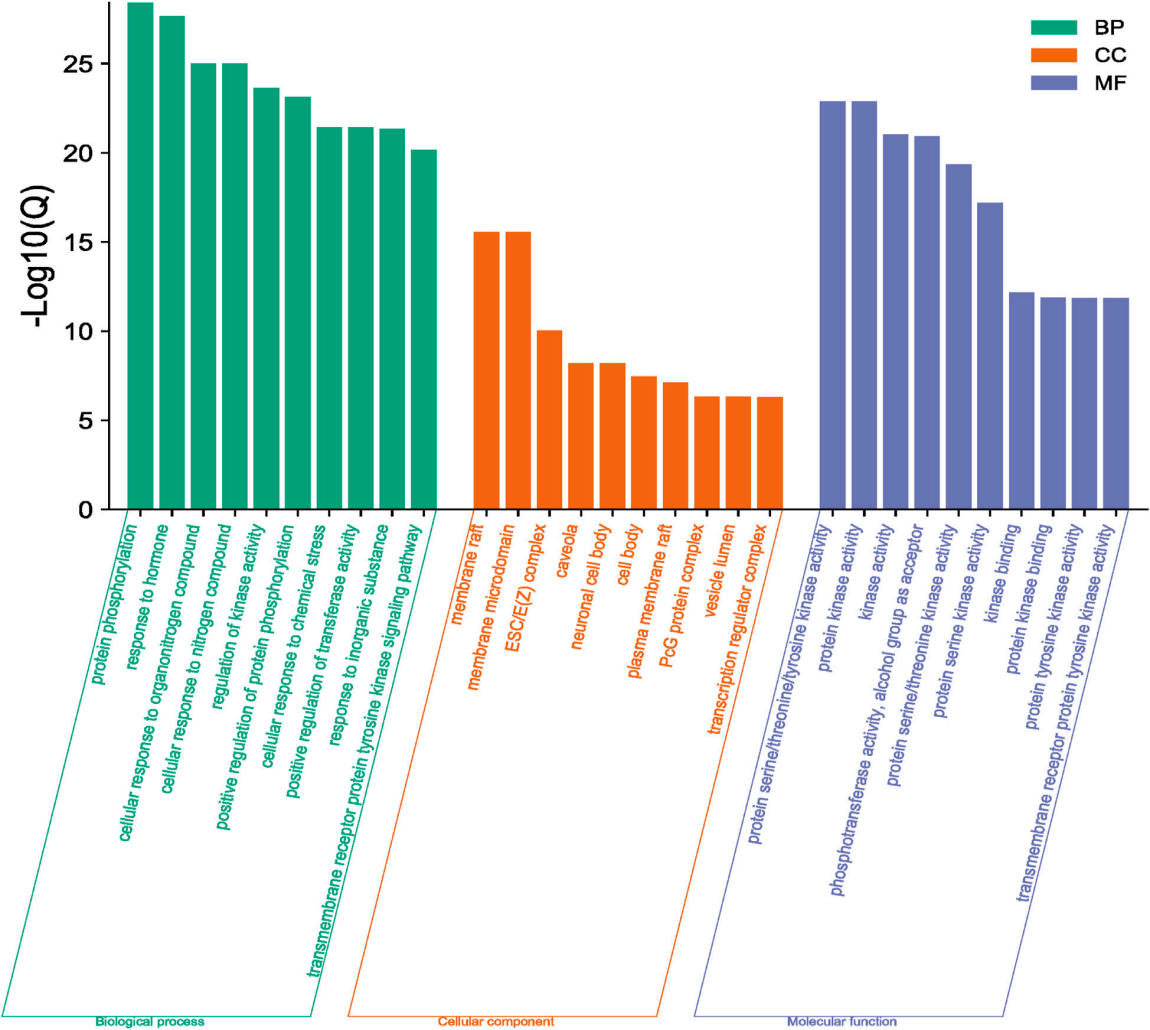
Results of GO enrichment analysis (Green column chart represents biological processes, orange column chart represents cellular components and purple column chart represents molecular functions).

**FIGURE 5:**
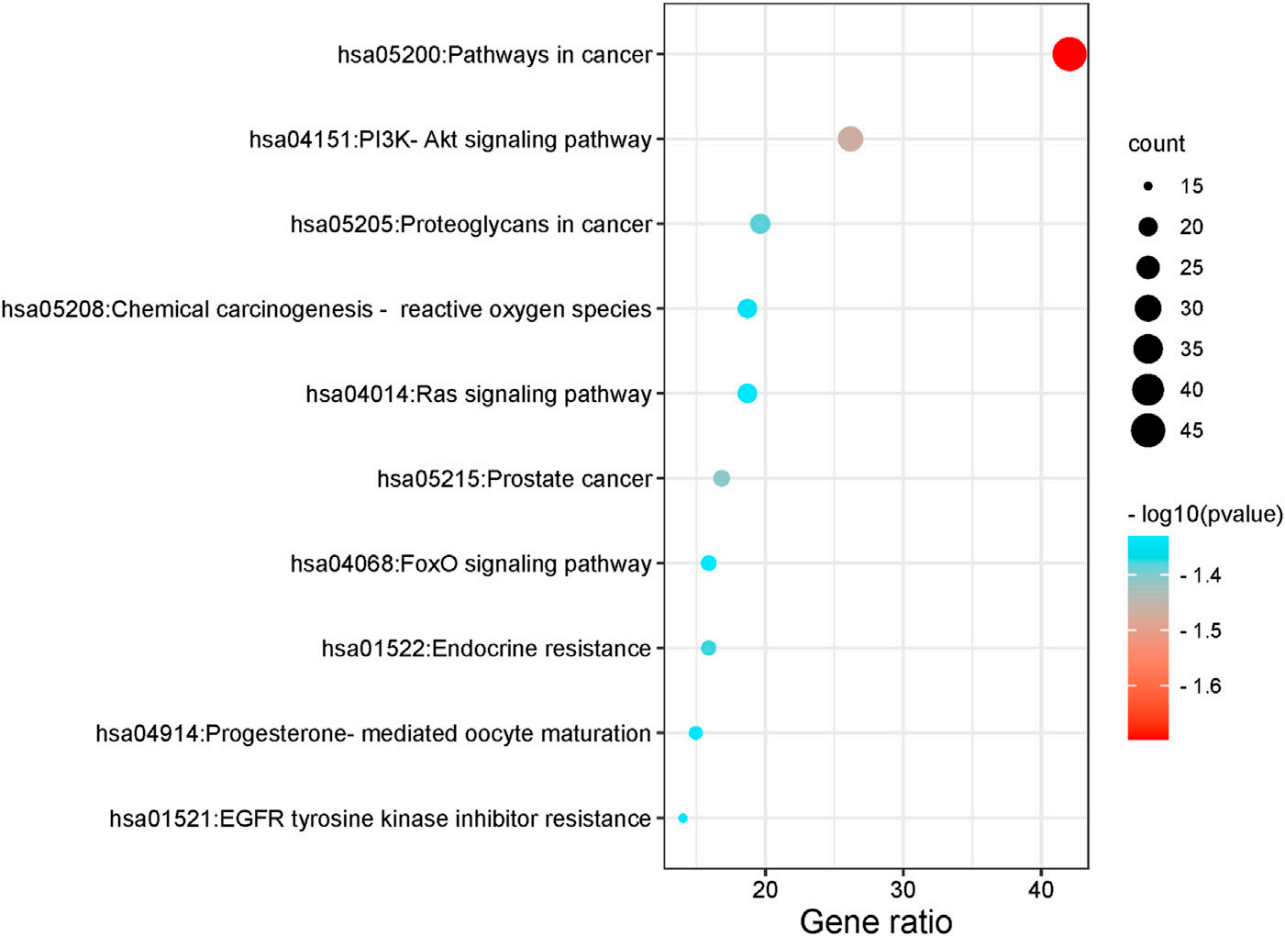
KEGG enrichment analysis results.

The functions and pathways of potential genes were analyzed by Metascape database to enrich information. The results of GO enrichment analysis are shown in Figure 4. With *p* < 0.001 as the screening condition, the first 30 GO items were screened, including 10 biological processes, 10 cellular components and 10 molecular functions. In addition, these items mainly focus on protein phosphorylation, response to hormones, membrane raft, kinase activity and so on. For KEGG pathway analysis, Table 2; Figure 5 list the first 10 related signaling pathways *p* < 0.001), which are mainly concentrated in proteoglycans in cancer, pathways in cancer, PI3K-Akt signaling pathways, endocrine resistance and so on.

**TABLE 2:** KEGG enrichment analysis and related genes.

Pathway	Genes	*p*-Value
Pathways in cancer	ABL1, AKT1, AKT2, XIAP, AR, BCL2L1, BMP2, CASP3, CCNA2, CCNE1, CDK2, CDK6, NQO1, EGFR, ESR1, FGFR1, GRB2, GSK3B, GSTM1, GSTP1, HDAC1, HDAC2, HMOX1, HSP90AA1, IGF1, IGF1R, IL2, JAK2, KIT, MDM2, MET, MMP2, MMP9, NOS2, PIK3R1, PPARG, MAPK1, MAPK8, MAPK10, MAP2K1, PTGS2, RARA, RARB, RXRA, TERT	<0.001
PI3K-Akt signaling pathway	AKT1, AKT2, BCL2L1, CCNE1, CDK2, CDK6, EGFR, ERBB4, FGFR1, GRB2, GSK3B, NR4A1, HSP90AA1, IGF1, IGF1R, IL2, INSR, JAK2, KDR, KIT, MDM2, MET, NOS3, PIK3CG, PIK3R1, MAPK1, MAP2K1, RXRA	<0.001
Prostate cancer	AKT1, AKT2, AR, CCNE1, CDK2, EGFR, FGFR1, GRB2, GSK3B, GSTP1, HSP90AA1, IGF1, IGF1R, MDM2, MMP9, PIK3R1, MAPK1, MAP2K1	<0.001
Proteoglycans in cancer	AKT1, AKT2, CASP3, MAPK14, EGFR, ERBB4, ESR1, FGFR1, GRB2, IGF1, IGF1R, KDR, MDM2, MET, MMP2, MMP9, PIK3R1, MAPK1, MAP2K1, PTPN11, SRC	<0.001
Endocrine resistance	AKT1, AKT2, MAPK14, EGFR, ESR1, GRB2, IGF1, IGF1R, MDM2, MMP2, MMP9, PIK3R1, MAPK1, MAPK8, MAPK10, MAP2K1, SRC	<0.001
Chemical carcinogenesis - reactive oxygen species	ABL1, AKT1, AKT2, CBR1, MAPK14, NQO1, EGFR, GRB2, GSTM1, HMOX1, MET, PIK3R1, MAPK1, MAPK8, MAPK10, MAP2K1, PTPN11, SOD2, SRC, AKR1C3	<0.001
EGFR tyrosine kinase inhibitor resistance	AKT1, AKT2, BCL2L1, EGFR, GRB2, GSK3B, IGF1, IGF1R, JAK2, KDR, MET, PIK3R1, MAPK1, MAP2K1, SRC	<0.001
Progesterone-mediated oocyte maturation	AKT1, AKT2, CCNA2, CDK2, MAPK14, HSP90AA1, IGF1, IGF1R, PDE3B, PIK3R1, PLK1, MAPK1, MAPK8, MAPK10, MAP2K1, AURKA	<0.001
Ras signaling pathway	ABL1, AKT1, AKT2, BCL2L1, EGFR, FGFR1, GRB2, IGF1, IGF1R, INSR, KDR, KIT, MET, PIK3R1, PLA2G2A, MAPK1, MAPK8, MAPK10, MAP2K1, PTPN11	<0.001
FoxO signaling pathway	AKT1, AKT2, CDK2, MAPK14, EGFR, GRB2, IGF1, IGF1R, INSR, MDM2, PIK3R1, PLK1, MAPK1, MAPK8, MAPK10, MAP2K1, SOD2	<0.001

### 3.5 Construction of Tan-IIA-target-NB network

**FIGURE 6:**
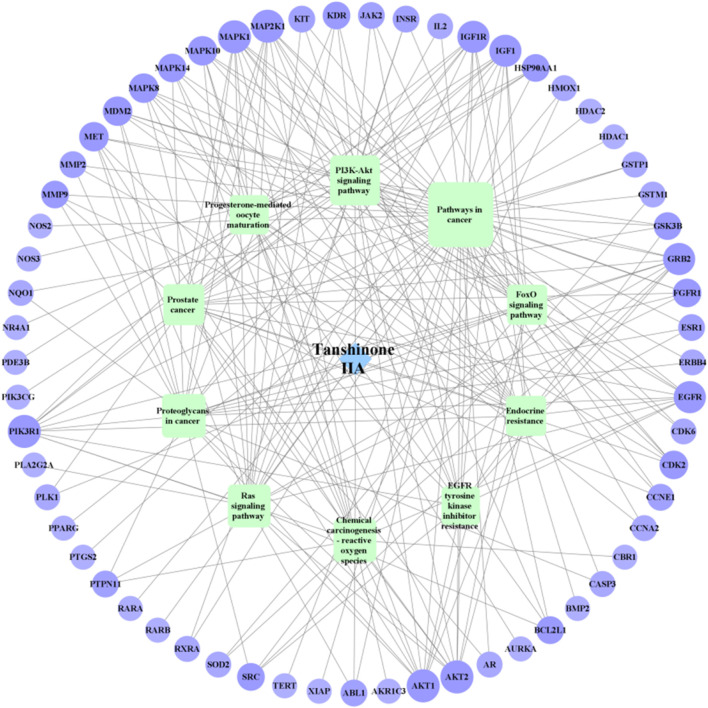
Tanshinone IIA-target-NB network (Blue diamond represents Tan-ⅡA, green square represents the KEGG pathway, purple circle represents the target, the figure size and the number of connections represent the importance of nodes).

**FIGURE 7:**
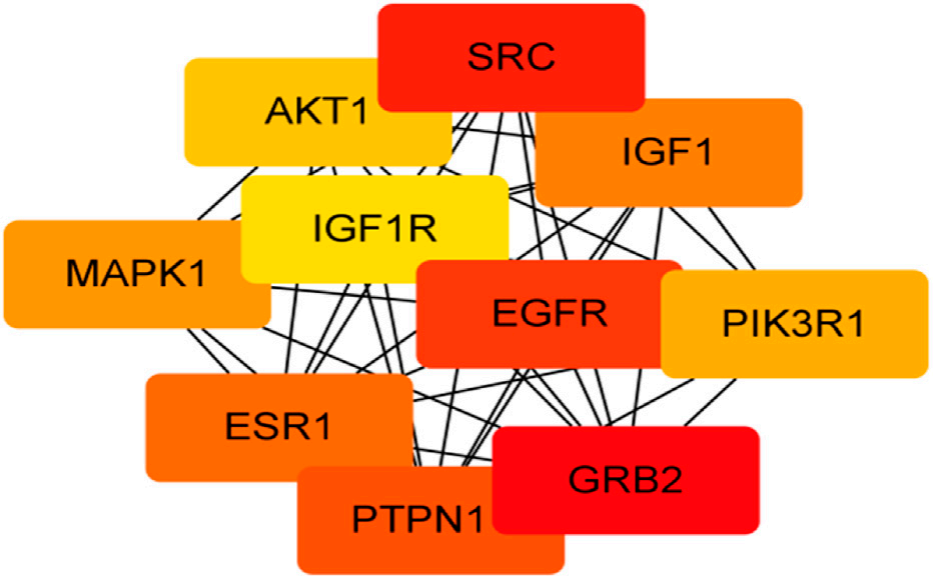
Visualization of the first 10 hub gene networks (Node color represents the node score, the darker and redder nodes mean the higher score).

For KEGG pathway analysis, Table 2; Figure 5 list the first 10 related signaling pathways *p* < 0.001), after deduplicating related targets, the Tan-IIA target pathway network contains a total of 72 nodes (Figure 6). Nodes of different colors and shapes represent different active compounds, targets and pathways, including 1 compound (blue diamond), 61 targets (purple circle) and 10 KEGG pathways (green square). The number of connections between nodes represents the importance of nodes in the network. At the same time, the first 10 hub genes are calculated using the cytoHubba plug-in in Cytoscape3.8.2 and visualized in Figure 7. As shown in Figure 7, the node color represents the node score, and the darker and redder nodes mean the higher score.

### 3.6 Molecular docking

Tan-IIA was docked with the first 10 Hub gene targets GRB2, SRC, EGFR, PTPN1, ESR1, IGF1, MAPK1, PIK3R1, AKT1 and IGF1R using AutoDockTools 1.5.6, and 10 groups of ligand-receptors were obtained. Results the binding energy between Tan-IIA and target protein were between-6.47∼-9.2 kcal/mol, the binding energy of each group was less than -5 kcal/mol, and the combination of less than -7 kcal/mol were found in 7 groups, accounting for 70%.

**FIGURE 8:**
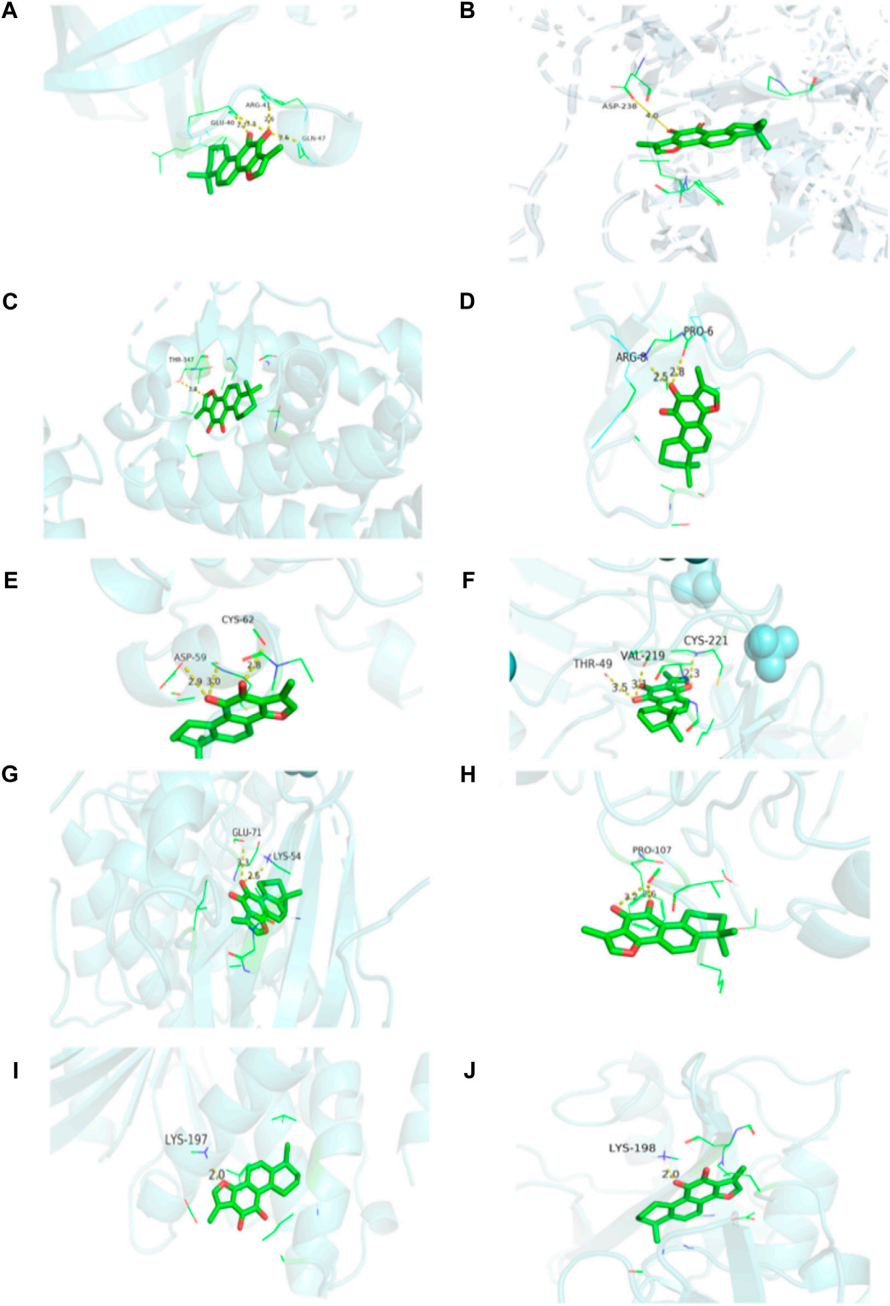
Visualization of protein-ligand interaction docking results. **(A)** The docking of Tan-IIA with AKT1; **(B)** The docking of Tan-IIA with EGFR; **(C)** The docking of Tan-IIA with ESR1; **(D)** The docking of Tan-IIA with GRB2; **(E)** The docking of Tan-IIA with IGF1; **(F)** The docking of Tan-IIA with IGF1R; **(G)** The docking of Tan-IIA with MAPK1; **(H)** The docking of Tan-IIA with PIK3R1; **(I)** The docking of Tan-IIA with PTPN1; **(J)** The docking of Tan-IIA with SRC. (yellow dotted lines represent hydrogen bonds).

According to the docking results, the binding energies of 10 target proteins to Tan-IIA were all less than -5 kcal/mol (Table 3) and were shown in docking mode 3D map (Figure 8). There are multiple hydrogen bonds between the core target protein and Tan-IIA (yellow dotted lines represent hydrogen bonds). As shown in Figure 8, Tan-IIA binds to the amino acid residues ARG-8 and PRO-6 sites of GRB2; LYS-198 sites of SRC, ASP-238 sites of EGFR; LYS-197 sites of PTPN1; THR-347 sites of ESR1; ASP-59 and CYS-62 sites of IGF1; THR-49, VAL-219 and CYS-221 sites of IGF1R; LYS-54 and GLU-71 sites of MAPK1; PRO-107 sites of PIK3R1; GLN-47, ARG-41 and GLU-40 sites of AKT1; CYS-221, VAL-219 THR-49 site binding.

**TABLE 3:** Docking information of Tanshinone IIA with 10 target proteins.

Gene	Binding energy	Gene	Binding energy
ESR1	−9.2	IGF1	−7.36
EGFR	−8.84	SRC	−7.09
IGF1R	−8.09	PIK3R1	−6.96
MAPK1	−7.79	AKT1	−6.93
PTPN1	−7.39	GRB2	−6.47

### 3.7 Tan-IIA inhibits the proliferation of SHSY-5Y

**FIGURE 9:**
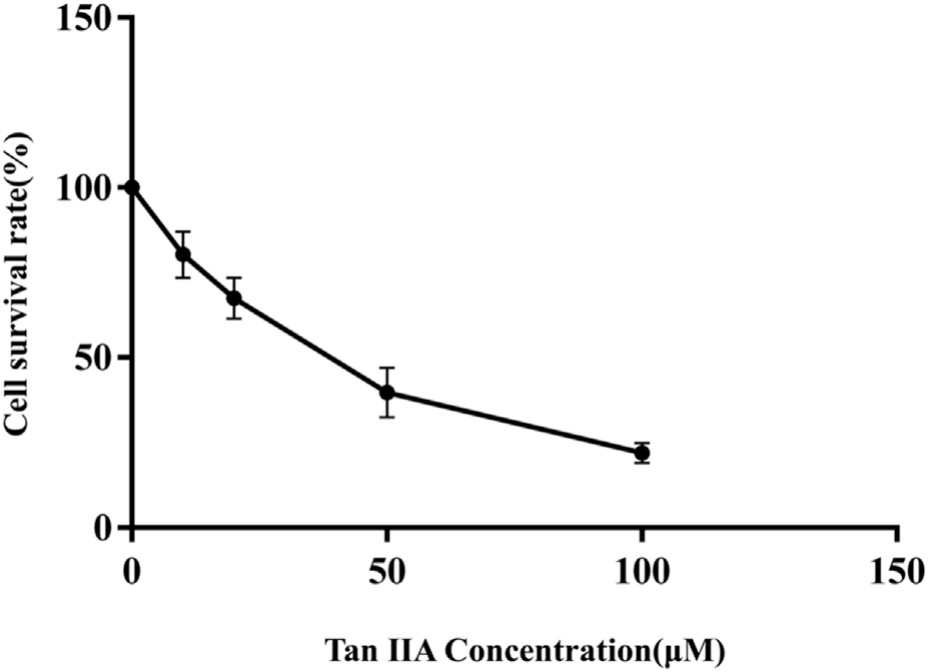
Viability inhibition of Tan ⅡA on SHSY-5Y under normal culture condition.

The effect of Tan ⅡA on the activity of human neuroblastoma SHSY-5Y cells was detected by MMT assays. Compared with control group, the survival rate of SHSY-5Y cells treated with different doses of Tan ⅡA (0, 20, 50 μmol L^-1^) for 24 h decreased with the increase of drug concentration, indicating that Tan ⅡA significantly inhibited the proliferation of human neuroblastoma SHSY-5Y cells. The 50% inhibitory concentration (IC_50_) of Tan ⅡA on SHSY-5Y cells determined by GraphPad Prism 9.0 was 34.98 μmol L^-1^ (Figure 9). Under the condition of Tan ⅡA intervention for 24 h, the concentration gradient (0, 20, 50 μmol L^-1^) was selected as the concentration of the follow-up experiment.

### 3.8 Tan-ⅡA inhibits SHSY-5Y migration

**FIGURE 10:**
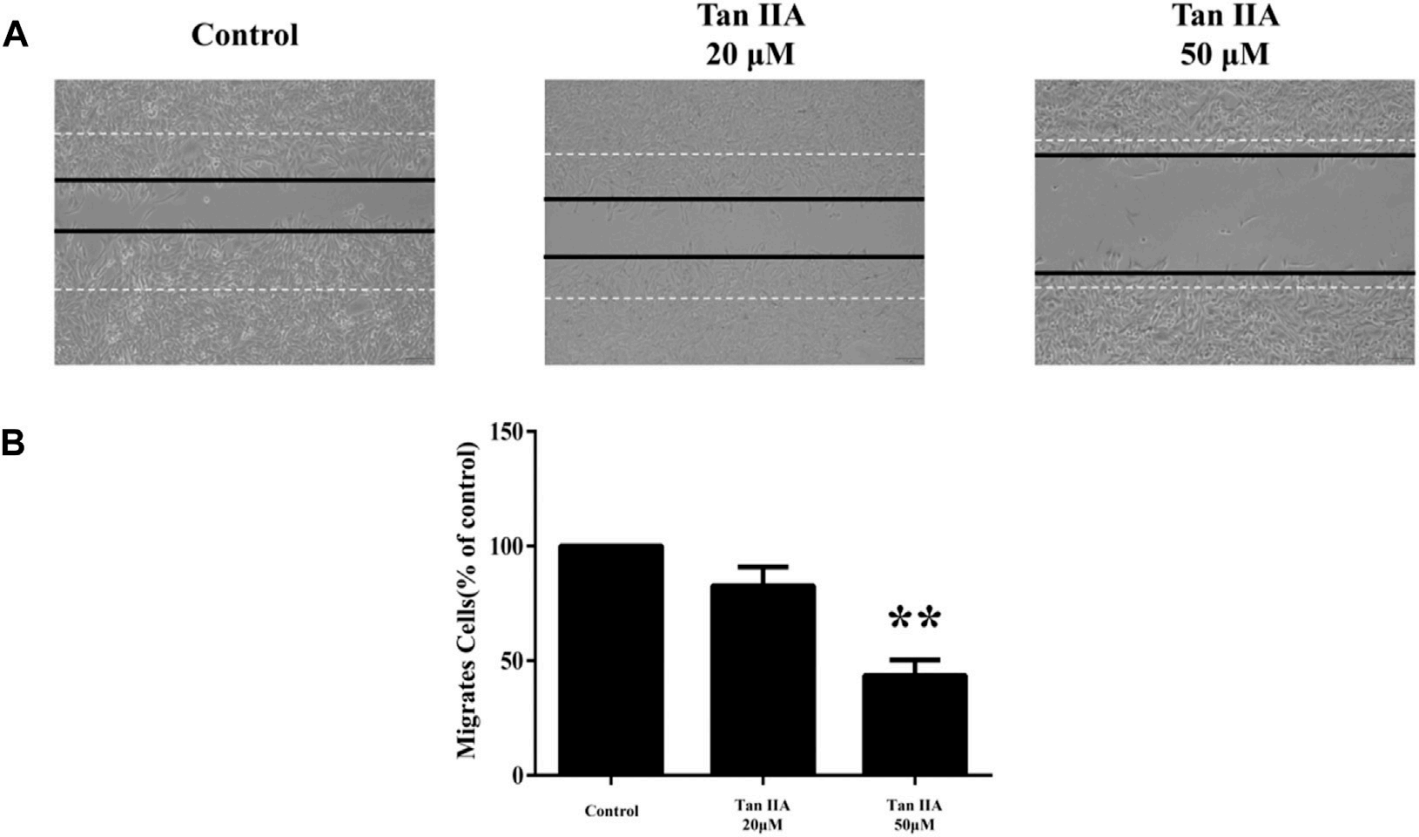
**(A)** Tan ⅡA inhibits SHSY-5Y migration; **(B)** Tan ⅡA inhibits SHY-5Y cell migration histogram. (**p < 0.01, Tan ⅡA 50 μM group vs. control group).

We carried out wound healing assays to investigate the effects of Tan ⅡA on cell migration to evaluate the ability of SHSY-5Y in the presence of various concentrations of Tan ⅡA (0, 20 and 50 μmol L^-1^). The results showed that Tan ⅡA could significantly inhibit the migration of SHSY-5Y in a dose-dependent manner (Figure 10).

### 3.9 Tan-IIA reduces lipid peroxidation of SHSY-5Y

**FIGURE 11:**
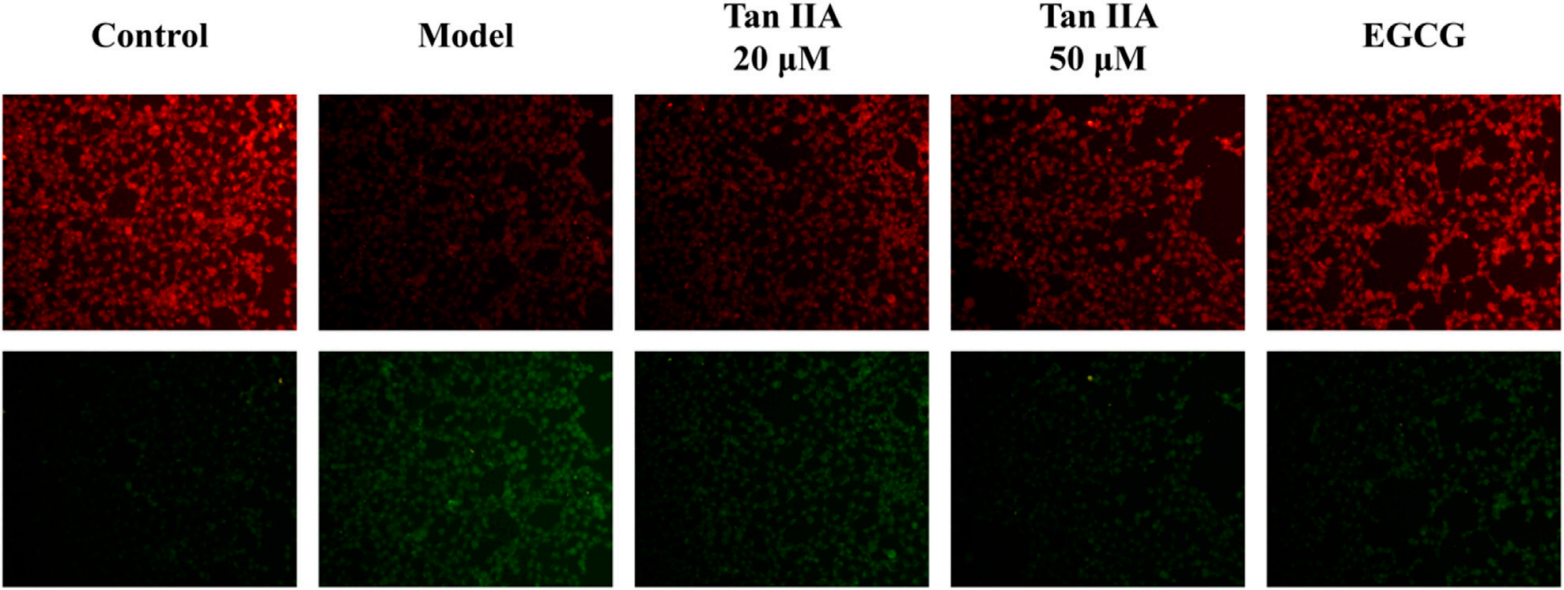
Tan ⅡA inhibits lipid peroxidation in SHSY-5Y cells.

BODIPY-C11 581/591 is commonly used to detect intracellular lipid peroxidation ROS levels. EGCG can reduce the incidence of cancer, neurodegenerative diseases induced by oxidative stress, diabetes and cytokine-induced inflammation *in vivo* (Yu et al., 2023). In addition, EGCG can significantly reduce the level of intracellular lipid peroxidation by reducing the activity of LDH and the level of ROS, increasing the content of NO and SOD and reducing lipid peroxidation, so it was chosen as the positive control (Orsolic et al., 2013). SHSY-5Y cells were treated with different concentrations of Tan-IIA (20, 50 μmol L^-1^) and EGCG (25 μmol L^-1^). The results in Figure 11 showed that the level of lipid peroxidation in the model group was significantly higher than that in the blank control group (*p* < 0.01). Compared with the model group, the level of lipid peroxidation in each treatment group was significantly lower than that in the model group (*p* < 0.05), the effect of 50 μM Tan ⅡA was similar to that of positive control drug EGCG (Table 4).

**TABLE 4:** Effect of Tan ⅡA on Lipid Peroxidation in SHSY-5Y cells.

	Control	Model	Tan ⅡA 20 μM	Tan ⅡA 50 μM	EGCG
Lipid peroxidation level	1.00	6.13 ± 0.21	4.26 ± 0.35	3.01 ± 0.41	2.97 ± 0.36

## 4 Discussion

Salvia miltiorrhiza is a kind of traditional Chinese medicine for promoting blood circulation and removing blood stasis. Recent studies have found that Salvia miltiorrhiza plays an important role in improving nervous system function and brain metabolism.

In this study, the network pharmacology and molecular docking methods were used to screen the mechanism of Tan-IIA in the treatment of human neuroblastoma. First of all, by combining multiple drug databases and disease databases, we initially obtained 107 overlapping targets (Figure 2) for the construction of PPI network, and we found that there were protein-protein interactions in 101 nodes (Figure 3). KEGG analysis showed that the pathway of Tan-IIA in the treatment of human neuroblastoma included Pathways in cancer、PI3K-Akt signaling pathway、Prostate cancer、Proteoglycans in cancer、Endocrine resistance、Chemical carcinogenesis - reactive oxygen species、EGFR tyrosine kinase inhibitor resistance、Progesterone-mediated oocyte maturation、Ras signaling pathway、FoxO signaling pathway and so on. It can be seen that these pathways are mainly related to anti-cancer, anti-apoptosis, antioxidant stress and other physiological and pathological processes (Liu et al., 2019), but also closely related to the pathological process of NB. Tan-IIA is associated with many disease targets in the anti-cancer and anti-tumor target network. Through PPI protein network analysis and Cytoscape software visualization, we can see that the key targets of Tan-IIA on human neuroblastoma are GRB2, SRC, EGFR, PTPN1, ESR1, IGF1, MAPK1, PIK3R1, AKT1, IGF1R and so on. GRB2 plays an important role in physiological processes such as cell proliferation and differentiation (Di Fulvio et al., 2007; Radtke et al., 2016). At the same time, it is highly expressed in many kinds of tumor tissues, such as breast cancer, leukemia, pancreatic cancer and so on (Daly et al., 1994; Feller et al., 2003; Yin et al., 2013), and can be located in the nucleus of tumor cells. SRC is a proto-oncogene, when a tumor occurs, SRC will show obvious distribution and shift in the cell, from cytoplasmic distribution to nuclear distribution. At the same time, it plays an important regulatory role in colon, breast, prostate, pancreatic and other tumor tissues (Summy and Gallick, 2003; Kasahara et al., 2007). EGFR, the epidermal growth factor receptor, is a member of HER family. EGFR is a receptor tyrosine kinase that participates in the regulation of cell growth, differentiation, survival and metastasis (Papanastasiou et al., 2017), Studies have shown that many tumorigenesis, such as lung cancer, lung adenocarcinoma and cholangiocarcinoma, are closely related to overexpression or mutation of EGFR (Sigismund et al., 2018; Thamrongwaranggoon et al., 2022). Similarly, the abnormal expression of PTPN1 in gastric cancer, ovarian cancer, colon cancer and other tumors can promote cell proliferation, colony formation and migration, while reduce the apoptosis of cancer cell lines (Feng et al., 2015; Yu et al., 2019), and the overexpression of PTPN1 is associated with poor prognosis (Leslie and Foti, 2011). ESR1 is a ligand-dependent transcription factor, which can mediate non-genotypic membrane signal pathways to enhance cell proliferation, which is not conducive to the survival of cancer patients. ESR1 is related to the occurrence of gynecological tumors such as breast cancer, endometrial cancer, uterine leiomyoma and so on (Yin et al., 2014; Gaillard et al., 2019). IGF1 is an insulin-like growth factor, which can promote scar formation and wound healing, regulate cell proliferation and metabolism, promote tumor cell growth, inhibit tumor cell apoptosis, and promote tumor occurrence and development (Javed et al., 2020). MAPK1 plays an important role in regulating the migration and growth of nerve cells, activating or overexpressing MAPK1 can promote the migration and invasion of tumor cells, increase cell viability and participate in epithelial-mesenchymal transformation (Noi et al., 2020; Wang et al., 2020). PIK3R1 is a potential protective target, which can negatively regulate the growth and invasion of tumor cells, and is closely related to the occurrence and development of tumor. The decrease of PIK3R1 is significantly correlated with tumor stage, metastasis and prognosis (Taniguchi et al., 2010; Abraityte et al., 2019). AKT1 plays an important role in the development, growth and metabolism of mammals. It has been found that the abnormal expression of AKT1 is related to schizophrenia, bipolar disorder, Parkinson’s disease and other diseases. Phosphorylated AKT1 is closely related to the growth, invasion, metastasis, apoptosis and autophagy of tumor cells (Howell and Law, 2020; Millischer et al., 2020; Dhahri et al., 2021). IGF1R is a tyrosine kinase that is overexpressed in various types of tumor cells and plays an important role in the proliferation and survival of tumor cells. At the same time, the expression of IGF1R in the heart plays an important physiological role in regulating cardiac contractile function and cell proliferation and differentiation (Bodzin et al., 2012; Pineiro-Hermida et al., 2017). GO enrichment analysis showed that the anticancer effect of Tan-IIA was related to plasma membrane, kinase activity and protein phosphorylation. The plasma membrane is a very thin membrane that surrounds the cell surface, which is mainly composed of membrane lipids and membrane proteins. The basic function of plasma membrane is to maintain the relative stability of intracellular microenvironment. These pathways play a crucial role in cell survival, proliferation, differentiation, and tumorigenesis.

The results of molecular docking of 10 hub genes screened by cytoHubba plug-in showed that Tan-IIA could effectively interact with 10 targets: GRB2, SRC, EGFR, PTPN1, ESR1, IGF1, MAPK1, PIK3R1, AKT1 and IGF1R. The results showed that they all had binding activity, and Tan-IIA had the highest binding or docking score to ESR1 (−9.2 kcal/mol). By analyzing the interaction mode between proteins and ligands, it can be concluded that Tan-IIA can bind these selected target proteins well and obtain lower binding energy mainly by forming hydrogen bonds. Molecular docking models can provide evidence for how compounds act on anti-cancer targets. The results of molecular docking preliminarily confirmed that Tan-IIA can play a therapeutic role in cancer through key targets.

Through the cell experiment, it was observed that Tan-IIA could effectively inhibit the proliferation of SHSY-5Y cells. In order to evaluate the effect of Tan-IIA on the survival of SHSY-5Y cells, its 24 h IC_50_ was determined to be 34.98 μmol L^-1^ by MTT experiment, which indicated that Tan-IIA could be a potential drug for the treatment of human neuroblastoma. In addition, scratch assay showed that Tan-IIA inhibited the migration ability of SHSY-5Y cells in a dose-dependent manner. Iron death represents a mode of programmed cell death that has been discovered in recent years, which is different from apoptosis. It is characterized by lipid peroxidation and iron accumulatio (Kajarabille and Latunde-Dada, 2019). Lipid peroxidation plays a key role in the initiation and progression of iron death. This process involves oxidative degradation of lipids driven by ROS, which eventually leads to cell damage and rupture of cell membrane. Lipid peroxidation and iron death promote each other, forming a complex interaction, in which ROS promotes iron accumulation, and the same iron accumulation enhances oxidative stress (Louandre et al., 2013). The level of intracellular lipid peroxidation was evaluated by BODIPY581/591C11 lipid peroxidation probe (Su et al., 2019). Iron death plays different roles in different stages of tumor development and inhibits tumor cell proliferation and differentiation in intracellular homeostasis regulation. Therefore, regulating iron death in human neuroblastoma has become a promising treatment strategy. Tan-IIA plays the role of anti-human neuroblastoma through multi-component, multi-target and multi-pathway, so it is an anti-tumor drug with high research value. This study provides a new insight into the possibility of iron death of human neuroblastoma cells induced by Tan-IIA, and provides a new mechanism for potential intervention in the treatment of human neuroblastoma, which needs further molecular function and clinical research.

## 5 Conclusion

In this study, the mechanism of Tan-IIA in the treatment of human neuroblastoma was studied systematically and comprehensively for the first time by means of network pharmacology and molecular docking. Drug target association prediction preliminarily analyzed the potential anticancer, anti-tumor targets and related signal pathways of Tan-IIA. 10 central genes and their related biological functions, as well as 10 main KEGG pathways were studied by network pharmacology. Ten potential targets for effective binding to Tan-IIA were obtained by molecular docking, which provides a basic theoretical basis for its anti-tumor research, basic experimental verification a basic basis for future experimental verification, and provides a better reference for the development and integration of anti-tumor research of traditional Chinese medicine and modern medicine. This study provides an idea for the research and development of new drugs for the treatment of human neuroblastoma and provides a theoretical basis for the clinical transformation of human neuroblastoma against human neuroblastoma.

## Data Availability

The original contributions presented in the study are included in the article/Supplementary material, further inquiries can be directed to the corresponding authors.
